# The Paradigm Shift from End of Life to Pre-Emptive Palliative Care in Patients with Cancer

**DOI:** 10.3390/cancers14153752

**Published:** 2022-08-01

**Authors:** Sebastiano Mercadante

**Affiliations:** Main Regional Center for Pain Relief & Supportive/Palliative Care, La Maddalena Cancer Center, Via San Lorenzo 312, 90146 Palermo, Italy; terapiadeldolore@lamaddalenanet.it or 03sebelle@gmail.com

**Keywords:** palliative care, supportive care, cancer

In most countries, health care providers have circumscribed palliative care in a network represented by home care and hospice care. This is equivalent to a short period, a few weeks before death [1], thus, assuming that palliative care is equivalent to end-of life care [2]. Although these services have a relevant role, they partially cover the needs of cancer patients requiring palliative care expertise. Of concern, this approach leads to late referrals when oncologic treatments are exhausted possibly after various and often inappropriate lines of chemotherapy, promoting just the compassionate aspects of palliative care.

In the last ten years, it has been clearly stated that cancer patients who access earlier palliative care have better clinical outcomes at potentially lower costs [3,4,5].

Indeed, a modern concept of palliative care suggests an expanding role in different settings and stages of disease. To provide a complete palliative offering, specialized palliative care should start in a comprehensive cancer center, where anticancer treatments are provided. Patients should be intercepted in their disease trajectory early [6]. Although different conceptual models of integration of oncology and palliative care have been proposed [7], the activation of a network starting in the hospital is not usual. Thus, the network activity should be anticipated, offering all the settings of palliative care, including the acute palliative care unit (APCU), outpatient clinic, internal and external consultations, hospice, and home care [8]. These pieces are complementary and not alternatives, as they should be chosen according to the individual needs in the different phases of disease trajectory in a circular way. For example, in the APCU, patients are prevalently admitted when they still receive anticancer treatments with a large variety of indications, including toxicity, clinical deterioration, psychological distress, pain, and other symptoms. Patients may also be re-evaluated to outline or to change the direction of care towards the transition to the best supportive care, allowing the withdrawal of active treatments. Hospice and home care should be promptly available and connected with the APCU to favor a continuity of care [9]. Patients who improve their conditions are able to undergo further treatments after a multispecialistic consultation. This crossroad has a fundamental role to preventing afinalistic treatments and to offer realistic alternatives. In these circumstances, experience in prognosis, communication, and breaking bad news is typical competence of a palliative care doctor [8]. Of interest, in recent years, oncologists have been referring patients who had not received any treatment before, requiring supportive therapies while waiting for a diagnosis or because they were considered incurable at the time of diagnosis. This constituted a sort of pre-emptive palliative care, preceding the oncologic treatment, expanding the concept of early or simultaneous palliative care [10]. Moreover, the hospital palliative care team may provide advice to other units, thus, influencing the decision-making process. For example, consultations in very intensive settings, such as surgery and onco-hematology, can prevent future disproportionate interventions, which can result in a further psychological and physical burden, with inacceptable increased costs. In such circumstances, a timely palliative care intervention may facilitate the communication with family members regarding the prognosis and allow to plan a proper treatment to limit patients’ suffering with a rapid transition of care.

An appropriate discussion with relatives about the short prognosis and the need to sedate for controlling suffering in the last hours of life is fundamental in preventing further suffering with intensive treatments [11]. Inappropriate admissions to intensive care units may be prevented, in favor of a palliative care setting. Consultations may be useful also when a deterioration and a lack of clinical response are observed in patients admitted to intensive care units. Family conferences for timely goal of care discussions are fundamental to make a decision to withdraw or withhold further futile treatments, and symptom control is mandatory during this process. Such decisions also help optimizing resources [12]. Additionally, long-survivor patients may require a pain or palliative care consultation ad libitum, due to the consequences of oncological treatments. Last, but not least, the hospital palliative care team also exerts a cultural role, exchanging their experience with colleagues, providing information for internal professionals or external visitors. Finally, a large volume unit offers an opportunity for high-quality research, due to the complexity and variability of patients.

Rather than generic “early palliative care”, a better term could be “timely palliative care”, which is personalized care based on patients’ needs and delivered at the optimal time and setting [13]. This all-in-one model has been spontaneously growing in our center, possibly due to a favorable context, long-term tradition, a certain scientific reputation, and relationships between leaders and local administration, regardless of the regional health care system, which instead still offers a delayed network of palliative care invariably based on hospice and home care.

Unfortunately such a model has not extensively expanded for different reasons in Italy as well as in other countries. Thus, different models have been suggested, but not all-in-one ones. The existing, different health care systems recognize palliative care in different ways with unbalanced economical resources. This results in conceptual differences and the fragmentation of services, each of them offering only a part that is often not connected with others, or simply only one option exists. The lack of palliative care physicians and proper leadership in hospitals is probably the most relevant issue. If there is a mobile team, a consultation or outpatient clinic are available, but this activity is often limited to a single-shot intervention, without connecting the patient to a network, allowing for a continuing pathway. Acute palliative care units are not available in most hospitals, including oncological departments. Patients with high levels of distress and complexity should be better admitted to these units for continuous and more specialistic assessments and interventions. Late referral by oncologists is another atavistic problem, also considering the number of available treatments often administered until the last weeks of life. A referral to palliative care is heterogenous and highly dependent on the individual clinician. Concerns have been raised regarding the lack of standard definitions for terminology in the supportive and palliative oncology literature [14]. Cultural attitudes, particularly in Mediterranean countries, where paternalism on behalf of family member is a rule, often rely on anticancer therapies rather than on palliative care, which are still seen as end-of-life care. The simple name change to supportive care, for example, was associated with more inpatient referrals and earlier referrals in the outpatient setting [15].

The lack of research activity is also dependent on the need for scientific leadership. On the other hand, as it occurs in many countries, including Italy, palliative care has been considered even by law to be offered only as home care or hospice care, which means a limited time to provide specialistic palliative care [2]. In a conceptual model, it has been suggested that supportive care services should be organized under one department with a unified approach to patient care, program development, and research [16].

Administrators of comprehensive cancer centers and health care providers should be aware of these barriers and of the need for an anticipated palliative care network to provide timely, proportionate, and meaningful intervention from the time of diagnosis to death or cancer recovery.
